# Next-Generation Sequencing Approaches in Cancer: Where Have They Brought Us and Where Will They Take Us?

**DOI:** 10.3390/cancers7030869

**Published:** 2015-09-23

**Authors:** Veronique G. LeBlanc, Marco A. Marra

**Affiliations:** 1 Genome Science & Technology graduate program, University of British Columbia, Vancouver, BC V6T 1Z4, Canada; E-Mail: vleblanc@bcgsc.ca; 2 Canada’s Michael Smith Genome Sciences Centre, BC Cancer Agency, Vancouver, BC V5Z 4S6, Canada; 3 Department of Medical Genetics, University of British Columbia, Vancouver, BC V6T 1Z4, Canada

**Keywords:** cancer, next-generation sequencing, translational research, precision oncology medicine

## Abstract

Next-generation sequencing (NGS) technologies and data have revolutionized cancer research and are increasingly being deployed to guide clinicians in treatment decision-making. NGS technologies have allowed us to take an “omics” approach to cancer in order to reveal genomic, transcriptomic, and epigenomic landscapes of individual malignancies. Integrative multi-platform analyses are increasingly used in large-scale projects that aim to fully characterize individual tumours as well as general cancer types and subtypes. In this review, we examine how NGS technologies in particular have contributed to “omics” approaches in cancer research, allowing for large-scale integrative analyses that consider hundreds of tumour samples. These types of studies have provided us with an unprecedented wealth of information, providing the background knowledge needed to make small-scale (including “N of 1”) studies informative and relevant. We also take a look at emerging opportunities provided by NGS and state-of-the-art third-generation sequencing technologies, particularly in the context of translational research. Cancer research and care are currently poised to experience significant progress catalyzed by accessible sequencing technologies that will benefit both clinical- and research-based efforts.

## 1. Introduction

Cancer research has witnessed unprecedented advances during the past decade, a good deal of which can be attributed to revolutionary next-generation sequencing (NGS) technologies and the data they provide. Though cancer was first proposed to be a genetic disease early in the 20th century [1], technological limitations restricted progress in identifying the underlying genetic causes of the disease for much of the next century. The past decade has witnessed the advent of NGS and, along with it, a significant increase in our understanding of cancer biology and the cooperative roles of the cancer genome, transcriptome, and epigenome. NGS has made large-scale projects such as The Cancer Genome Atlas (TCGA) [2] and the International Cancer Genome Consortium (ICGC) [3] feasible, providing researchers with multi-platform data for thousands of tumours from a variety of cancer types and subtypes. This has in turn fuelled an expansion of translational research where data obtained from patient samples inform both research and clinical care. Recent case studies demonstrate the utility of whole-genome and -transcriptome sequencing for informing treatment decisions as well as providing valuable research insights. Indeed, these case studies provide excellent examples of the potential of precision (or personalized) oncology medicine, wherein a tumour is characterized at the genomic and/or transcriptomic levels in order to inform treatment. In this review, we will provide an overview of the contributions to cancer research provided by NGS technologies, including rapidly evolving third-generation technologies such as single molecule, real-time (SMRT) sequencing. We will also discuss current translational research efforts and how large-scale, multi-platform integrative analyses have provided us with the background knowledge needed to inform small-scale (including “N of 1”) studies, allowing us to begin taking a precision approach to cancer research and treatment.

## 2. A Brief History of Sequencing Approaches in Cancer

Throughout the 20th century, groundbreaking and painstaking studies provided insights into the linkages between genes and cancer initiation, development, and aggressiveness. For example, Nowell and Hungerford [4] used cytogenetic methods in 1960 to first describe the Philadelphia chromosome, which underpins chronic myeloid leukemia (CML); however, thirteen years elapsed before the Philadelphia chromosome was identified as the product of a translocation between chromosomes 9 and 22 [5], and the translocation was finally revealed to cause a fusion between the *BCR* and *ABL* genes in 1985 [6]. The fusion protein is a constitutively active tyrosine kinase, and its discovery led to the development of small-molecule tyrosine kinase inhibitors (TKIs) [7]. Though TKI therapy is usually not curative, its introduction revolutionized CML therapy and recent developments continue to improve the standard of care [7].

Technological advances continued to allow for the identification of a wide range of cancer-related genetic anomalies. Cytogenetic techniques, such as fluorescence *in situ* hybridization (FISH) and comparative genome hybridization (CGH), helped identify several additional recurrent chromosomal aberrations and revealed important aspects of cancer biology (e.g., identification of *PIK3CA* as an oncogene in ovarian cancer [8]). In addition, early sequencing technologies developed during the same period by Ray Wu [9,10,11] and Frederick Sanger [12,13] allowed for the targeted sequencing of specific regions and therefore helped identify recurrent mutations in genes of interest (e.g., hotspot mutations in the *TP53* gene [14]). In the 1990s, the development of DNA microarrays provided the first opportunity to look at genes on a larger scale. Indeed, oligonucleotide probe arrays (“DNA chips”) provided researchers with a radical new tool capable of providing readouts for many genes in parallel, in contrast to single-gene approaches [15,16]*.* Chip technology was first applied to human cancer cells in 1996 in an experiment measuring gene expression in the melanoma cell line UACC-903 with and without re-introduction of a normal human chromosome 6, which suppresses the tumourigenic properties of the cell line [17]. The authors were able to show that introduction of the normal chromosome was associated with differential expression of several genes potentially associated with the melanoma phenotype. Soon after, microarrays were used to demonstrate that cancers could be classified based on their gene expression profiles, specifically demonstrating differences between acute myeloid leukemia (AML) and acute lymphoblastic leukemia (ALL) [18].

In 1990, just five years after Sanger’s chain termination sequencing method was partially automated for the first time [19], the Human Genome Project was launched as a collaboration between genome centres in several countries [20]. A draft sequence was published eleven years later [20], and the project was deemed completed in 2004 [21]. The first phases of the project saw important technological advances, such as the development of improved dye-labeled terminators [22] and thermostable DNA polymerases [23]. Along with the introduction of capillary sequencing [24], these innovations greatly facilitated the completion of the human genome sequence. Advances in automation, such as improved base- and genotype-calling programs [25,26,27], also helped improve upon the speed of both sequencing and subsequent sequence analysis, setting the stage for further technological advances.

## 3. Current Sequencing Technologies

### 3.1. Second-Generation Technologies

The Human Genome Project and its accompanying need for large-scale sequencing approaches and data analysis inspired the creation of NGS methods, which allowed for DNA fragments to be sequenced in a massively parallel fashion. The number of sequence reads obtained using NGS was orders of magnitude higher than that obtained by capillary electrophoresis-based Sanger sequencing; however, this was achieved at the expense of both read length and accuracy. The first commercially available next-generation sequencer provided an ~100-fold increase in throughput relative to contemporary Sanger sequencers; however, read lengths were ~100 base pairs (bp) compared to the ~700 bp read lengths provided by capillary sequencing [28]. Sequencing platforms from different manufacturers were in relatively wide use by 2007 [29], and many improvements in these technologies and the emergence of new ones have subsequently occurred over the past decade. Nevertheless, read lengths provided by second-generation sequencing (SGS, also referred to as short-read sequencing) platforms in use today range from ~100–500 bp [30]. A concise review of these platforms and their sequencing process can be found at [30]. Importantly, NGS technologies also present an exponentially higher computational burden than previous technologies due to their massive increase in throughput [31]. Interpretation of NGS results is particularly complex in the context of cancer research, where a wide range of genomic aberrations can be of interest, and where some alterations may only exist in a subset of tumour cells [32]. Bioinformatics approaches are continuously evolving, and many applications are being developed that help non-bioinformaticians navigate NGS-derived data [33].

### 3.2. Third-Generation Technologies

#### 3.2.1. SMRT Sequencing

So-called third-generation sequencing (TGS) technologies have recently begun to emerge. This newest generation of sequencing methods is generally composed of technologies that interrogate single DNA molecules instead of clusters of DNA templates, thereby offering several advantages over SGS approaches, such as the elimination of amplification biases [34,35] (Table 1). One of the most widely used TGS technologies is SMRT sequencing, first developed in 2009 [36]. One of the main advantages of SMRT sequencing lies in its ability to produce unusually long read lengths; indeed, average read lengths have been shown to reach 21 kilobases (kb), and continue to improve with the introduction of new reagent kits [37,38]. These read lengths thus allow for the resolution of complex and repetitive genomic regions for a fraction of the time and cost needed for resolution by Sanger sequencing [39]. For instance, high-fidelity long PCR and SMRT sequencing were used to resolve a complex, highly repetitive central exon of the *MUC5AC* gene [40], which has been implicated in colorectal [41] and pancreatic [42] cancer. Advantages provided by SMRT sequencing come at the cost of higher error rates, most often due to insertions and deletions (indels); however, these errors are generally not context-specific, and methods and software that help reduce their impact have been introduced (e.g., the Quiver consensus algorithm [43]). The technology is also biased towards the identification of long fragments, with a recent study showing that novel transcript isoforms less than 300 bp in length identified by short-read sequencing were generally not validated by SMRT sequencing [44]. To address this and other caveats, several groups have shown that a combination of SMRT and short-read sequencing, termed hybrid sequencing, can provide highly accurate sequence results, especially for complex genomic regions [45,46,47] and transcript isoforms [48,49,50,51].

Though SMRT sequencing is often used for the study and assembly of small genomes, such as bacterial genomes [52], the long read lengths are also well suited for sequencing large human cancer-related loci, such as gene fusions products. For instance, a recent study identified *TTYH1*-C19MC fusions driving the expression of the microRNA cluster C19MC in embryonal tumours with multilayered rosettes (ETMRs) [53]. The authors showed that this in turn led to over-expression of an embryonic, brain-specific *DNMT3B* isoform, prompting them to propose a novel model of tumourigenesis for this cancer type. Similarly, two recent studies were able to confirm the presence of kinase domain mutations within *FLT3* genes with activating internal tandem duplications (*FLT3-ITD*) [54,55], which are found in ~20% of AML patients and are associated with poor prognosis. SMRT sequencing has also been used to sequence the entire BCR-ABL1 fusion gene transcript, allowing for the detection of compound mutations and splice isoforms [56]. The authors suggest that such an assay would be beneficial in the clinical setting, where mutations that confer resistance to TKI-based therapy could be readily identified. Though 454 sequencing had previously been used to address this issue, read lengths are not sufficient to cover the entire transcript, introducing the possibility of amplification biases affecting the measurement of mutation frequencies [57,58]. SMRT sequencing has also been applied to the detection of other structural variants, such as deletion and translocation breakpoint determination [59]. Another advantage presented by SMRT sequencing lies in its innate ability to directly detect and differentiate between base modifications such as 5mC and 5hmC (discussed in more detail below) as a consequence of these base modifications uniquely affecting the output of the method [60].

**Table 1:** Advantages and limitations of sequencing technologies.

Technology	Advantages	Limitations
Sanger sequencing	Long reads (~700 bp) High accuracy	Low throughput
Second-generation sequencing	High throughput	Short reads (~100–500 bp) Amplification biases generally occur
Third-generation sequencing	Long reads (average length can reach ~14 kb) High throughput No amplification needed Can detect and differentiate between base modifications Potential for miniaturization of the technology (nanopore sequencing)	High error rate Biased towards long fragments

#### 3.2.2. Nanopore Sequencing

More recently, nanopore-based sequencing technologies have also emerged as promising single-molecule sequencing strategies. Oxford Nanopore, who is leading the development of this technology, released its portable MinION sequencer to a select community of researchers for testing as part of the MinION Access Program (MAP) [61]. The novel sequencing device is USB-powered, allowing for sequencing runs to be performed on a consumer grade computer and thereby greatly increasing both portability and ease of sequencing. In addition, the company promised that the device would cost only $1000 and provide read lengths orders of magnitude longer than existing technologies. The platform was released in 2014, and preliminary reports suggest that the technology, while promising, requires further improvement [61,62]. Improvements in the chemistry have already led to advances in read quality [62]. The concept of a truly portable high-throughput sequencing platform is attractive in several applications, including fieldwork and point of care diagnostics [61,62,63,64], but significant advances are apparently required to fabricate such a device.

## 4. A Next-Generation “Omics” Approach to Cancer

### 4.1. The Genome

NGS has provided us with powerful access to the human genome, and has laid the groundwork for the field of cancer genomics. Indeed, given that cancer is fundamentally a genetic disease, NGS was quickly applied to study cancer genomes. In 2008, two groups published full human genome sequences obtained using NGS technologies [65,66]. In the same year, the first cancer genome and the first tumour/normal genome comparison were published in a study that used data from AML tumour cells and matched normal cells [67]. The study identified ten genes harbouring acquired mutations in the tumour, only two of which had been previously described in AML. The study established that whole-genome sequencing of tumour cells allowed for the identification of cancer-associated mutations, heralding a revolution in cancer research in which similar analyses across an expanding number of tumours and cancer types have been published.

Today, NGS is used in both clinical and research settings. Targeted genetic tests are currently used as diagnostic and prognostic tools in clinical oncology, and more extensive genomic tests seem likely to come into regular use in the near future [68,69]. Targeted cancer panels are advantageous due to their low cost and relatively simple interpretability, and many exist both for specific cancers, such as prostate cancer [70], and for more general application, such as solid tumours [71]. Conversely, whole-genome sequencing (WGS) is more frequently used in the research setting in order to obtain a complete and relatively unbiased view of the genome. Nevertheless, WGS incurs a high sequencing cost and computational burden due to the amount of data produced, and alternate approaches such as whole-exome sequencing (WES) are also used for certain applications [72]. WGS and WES of tumour and matched normal cells allows for the identification of acquired somatic mutations in the tumour, even those that occur at a relatively low frequency if sufficient depth of coverage is achieved [72]. Importantly, WGS requires no *a priori* selection of genes to be profiled, such as the specification of probe sequences often needed for microarray analyses, allowing for the identification of novel alterations. Indeed, WGS provides an unparalleled global view of the genome, allowing for the identification of somatic mutations even in non-coding and unannotated regions of the genome. High-coverage genomic data also allows for the detection of chromosomal rearrangements [73] and for high-resolution identification of copy number variants [74]. The identity and distribution of these and other types of genomic alterations vary widely between cancer types; indeed, patterns of mutations, termed mutational signatures, can be indicative of the underlying mutational processes and are often similar within a cancer type or subtype [75]. Importantly, genetic variants are not generally found uniformly throughout a single tumour; rather, the subclonal distribution of the variants means that they are typically found in 5%–50% of reads obtained by WGS [76]. Though genetic heterogeneity within tumours was recognized before the advent of NGS [77,78], both deep sequencing (*i.e.*, high- or ultra high-coverage) and single-cell sequencing have greatly improved our ability to study this phenomenon [76]. In 2011, Navin *et al.* [79] published the first report of single-cell sequencing in the context of tumour evolution, identifying three distinct subclonal populations within a genetically heterogeneous breast cancer tumour. Analysis of a primary breast tumour and its liver metastatsis also suggested that the primary tumour was composed of a single clonal expansion, which also seeded the metastasis. Notably, the sparse coverage obtained by single-cell sequencing only allowed for inferences to be made based on copy number profiles; however, the authors combined data from 100 cells for each tumour, and presented results that were consistent with previous studies suggesting that metastatic cells arise late in tumour development. Though single-cell genomic studies are becoming more frequent, this technology remains to be improved in order to overcome its current limitations, such as whole-genome amplification biases and the prohibitive cost of high sequence coverage [80,81].

The Catalogue of Somatic Mutations in Cancer (COSMIC) is a manually curated database composed of single nucleotide variant (SNV, in both coding and non-coding regions), indel, gene fusion, genome rearrangement, copy number, and differential expression data from over 12,000 cancer genomes [82]. Approximately half of the COSMIC data is obtained from TCGA and ICGC, and is representative of the power supplied by such large-scale studies to identify recurrent genomic events that participate in cancer initiation and progression. Recent studies using hybrid sequencing have also identified novel structural alterations not previously detected by traditional WGS [51,83]. Further technological advances will undoubtedly continue to reveal additional genomic alterations involved in cancer pathology, and such databases are bound to continue growing.

### 4.2. The Transcriptome

#### 4.2.1. Gene Expression

In addition to genome characterization, NGS has also been deployed to characterize the cancer transcriptome through RNA sequencing (RNA-seq). This is particularly useful in the context of cancer, a disease characterized by global genomic dysregulation. RNA-seq allows for the quantification of messenger RNAs (mRNAs), providing a measure of gene expression, and can also be used to uncover genome-level alterations as well as novel, disease-associated transcripts and transcript modifications [84]. Indeed, Meyer *et al.* [85] have identified mRNA transcripts containing *N*^6^-methyladenosine (m^6^A), and have shown differences in the levels of this modification in cancer cell lines. Additionally, high RNA-seq coverage allows for the identification of intragenic fusions that may not be identifiable using WGS, including in-frame fusion events that result in the activation of an oncogene [86]. For instance, novel and recurrent kinase gene fusions were recently identified through an analysis of RNA-seq datasets from 12 different cancer types [87]. The study uncovered new fusion partners, such as *TBL1XR1*-*RET* fusions in thyroid cancer, as well as known fusions in new cancer types, such *EGFR*-*SEPT14* fusions in low-grade gliomas, typically found in glioblastoma. The authors suggest that current protocols for targeted genomic profiling of patients would benefit from improvements that allow them to detect such gene fusions, which can in some cases be therapeutically actionable. RNA-seq is also able to detect transcribed somatic mutations [88], as well as isoforms created by alternative splicing events [89,90]. Alternative splicing has been shown to play an important role in tumorigenesis, and can in some cases be a target of therapy (reviewed in [91]). However, short-read sequencing remains limited in its ability to characterize mRNA isoforms, and complex eukaryotic transcriptome analysis will benefit from emerging long-read sequencing technologies that are able to encompass entire transcripts [92]. Indeed, Au *et al.* [50] used hybrid sequencing to characterize human embryonic stem cells (hESCs) and identified thousands of isoforms, including nearly 300 RNAs from novel gene loci.

Nevertheless, gene expression profiling generally remains the most common application of RNA-seq. NGS allows for the quantification of thousands of gene transcripts, and has led to improvements in cancer classification systems, providing enhanced diagnostic, prognostic, and therapeutic criteria. For instance, the IntClust classification system, based on the expression of driver genes, groups breast cancers into clinically and biologically valid subtypes and is better able to explain expression patterns reported by TCGA than the traditional PAM50 classifier [93,94]. Gene expression, either alone or in combination with mutational data, can also be used to investigate spatial and temporal tumour heterogeneity. For instance, several groups have recently analyzed both inter- and intra-tumour heterogeneity in glioblastoma multiforme (GBM), both of which are highly prevalent and pose serious challenges to the diagnosis and treatment of the disease (for a review, see [95]). Patel *et al.* [96] performed single-cell RNA-seq on 430 cells from five primary GBM tumours and found that each tumour contained a mixture of cells whose profiles correspond to established GBM subtypes. Cells representative of the proneural subtype, for example, were found in all five tumours, regardless of the tumour’s dominant subtype. Importantly, the authors suggest that, though established population-level GBM taxonomy has prognostic significance, it does not reflect the transcriptional diversity found within the tumour. Given that this diversity has the potential to confound therapeutic strategies, bulk tumour analyses of highly heterogeneous tumours may not prove sufficient to inform targeted treatment decisions.

In 2014, Tilgner *et al.* [48] published the first personal, allele-specific transcriptome using hybrid sequencing. With this technology, the authors were able to identify novel splicing isoforms, as well as to assess differential allelic expression and isoforms by linking RNA molecules to personal variants on a one-by-one basis. Such in-depth analyses can evidently provide valuable insights into the biology of individual tumours, and may one day be used to guide individualized treatment. However, single-cell genomics and transcriptomics present their own computational challenges, and further advances will be needed before these technologies reach their full potential [80,81]. Given that genomic mutations alone cannot fully explain tumour biology, both individual and large-scale transcriptomic analyses can help improve our understanding of the origin, pathogenesis, and aggressiveness of individual cancers, as well as characterize similarities and differences both within and between cancer types.

#### 4.2.2. Non-Coding RNAs

Though only ~2% of the genome encodes for genes, the Encyclopedia of DNA Elements (ENCODE) has found that ~75% of the genome is transcribed into primary transcripts [97]. Non-coding RNA species such as microRNAs (miRNAs) and long non-coding RNAs (lncRNAs) play important roles in a variety of cellular processes, and have been shown to be widely dysregulated in cancer [98]. MiRNAs are short single-stranded RNA molecules ~22 bases in length that can regulate the expression of target genes through translational repression or mRNA degradation [99]. A single miRNA can regulate the expression of several genes [100], and estimates suggest that up to ~60% of protein-coding genes are targets of miRNA regulation [101]. Unsurprisingly, a large number of miRNAs can thus act as tumour suppressors or oncogenes and exert widespread gene- and pathway-level effects, and their dysregulation is a central event in many cancer types [102]. MicroRNA-31 (miR-31), for instance, has been implicated as a tumour suppressor and an oncogene in different cancer types, and has recently been shown to play a role in cell cycle and epithelial to mesenchymal transition (EMT) regulation in hepatocellular carcinoma [103]. A recent study also showed that miRNAs with abundant expression in diffuse large B-cell lymphoma (DLBCL) regulate the expression of genes involved in metabolism, cell cycle, and protein and chromatin modification [104]. The authors also identified both known and candidate novel miRNAs whose expression is significantly associated with survival. By virtue of their size, miRNAs can be easily isolated and converted to small RNA cDNA libraries, which can then be subjected to massively parallel sequencing (miRNA-seq) [99]. Though microarrays and reverse transcriptase quantitative PCR (RT-qPCR) can be used to quantify miRNA expression, NGS technologies allow for large-scale characterization of both known and novel miRNA species [99]. Indeed, miRNA-seq has been applied to all TCGA datasets except for GBM [105], demonstrating both the power of the technology and the relevance of miRNA profiling in cancer.

Long non-coding RNAs (lncRNAs), though more poorly characterized than miRNAs, are also increasingly recognized as playing an important role in oncogenesis and cancer pathology. Different lncRNAs can serve diverse functions, such as guiding the site specificity of chromatin-modifying complexes (e.g., XIST and HOTAIR, discussed in more detail below) or acting as regulators of protein signalling pathways involved in carcinogenesis (e.g., lincRNA-21) [106]. The latter instance is particularly interesting in the context of p53, whose transcriptional network includes at least 18 lncRNAs that act in a positive regulatory feedback loop to enhance p53’s tumour suppressor activity [107,108]. LincRNA-21, for instance, is activated by p53 and also serves as an effector of p53 signalling through repression of target gene expression, thereby participating in p53-mediated apoptosis [109]. NGS, especially compared to earlier techniques, provides a relatively comprehensive and balanced view of a cell’s transcriptome, including previously unannotated species. Traditional RNA-seq includes a library preparation step that selects for polyadenylated transcripts, thereby allowing for the characterization of almost all mRNAs and the majority (~75%) of lncRNAs [110]. Though non-polyadenylated transcript libraries can be constructed [110,111], a recent study has shown that both RNA extraction and library selection methods affect the amount of intronic transcripts included in the library [112]. The authors propose a double selection method that can effectively be used to characterize both polyadenylated and non-polyadenylated transcript fractions. Overall, non-coding RNA studies have greatly benefited from NGS technologies, which allow for the quantification and characterization of both known and novel RNA transcripts. However, ncRNAs are generally expressed to a lower level than mRNAs, and thus require higher coverage to be reliably detected [92]; therefore, ncRNA studies will also benefit from further technological advances that will allow for higher depth of coverage at a lower cost.

### 4.3. The Epigenome

#### 4.3.1. DNA Modifications

Though cancer is fundamentally a genetic disease, it is also characterized by widespread epigenetic changes, of which aberrant DNA methylation was the first to be recognized and remains one of the most intensively studied. In humans, DNA methylation almost exclusively occurs at the 5ʹ position of a cytosine ring within a CpG dinucleotide [113]. These dinucleotides are found at a lower frequency than would be expected by chance throughout the genome, and typically cluster together in regions termed CpG islands (CGIs) [113]. Feinberg and Vogelstein [114] first reported in 1983 that gene loci could be hypomethylated in tumour cells compared to normal cells. The CpG island methylator phenotype (CIMP), which consists of genome-wide CpG island hypermethylation, was first described more than a decade later [115]. Though originally identified in colorectal cancer, CIMP-positive tumours occur in several cancer types and often constitute a unique subtype (for a review, see [116]). In general, a high proportion of tumours exhibit widespread changes in DNA methylation patterns, most often including global hypomethylation and regional hypermethylation [117]. For instance, the expression of caveolin-1 (*CAV1*), a potential oncogene involved in breast cancer pathogenesis, has been shown to be regulated by differential methylation of CGI shores, which are regions of lower CpG density that flank CGIs [118]. The authors highlight a negative correlation between the survival rate of estrogen receptor α (ERα)-positive breast cancer patients and *CAV1* expression, suggesting that *CAV1* CGI shore methylation could be used as a prognostic marker for this type of cancer. Studies such as this one reaffirm the usefulness of interrogating the whole genome (or a relatively loosely targeted region of the genome), as relevant changes can occur in regions that were not previously of interest.

Though many methods exist to measure 5-methylcytosine (5mC), the most common involve exposure of DNA to sodium bisulphite, which allows for unmodified cytosine residues to be converted to uracil while 5mC remains stable [119]. Following subsequent PCR amplification, originally unmodified cytosine residues are read as thymine residues, whereas original 5mC residues are read as cytosine residues [119]. Whole-genome bisulphite sequencing (WGBS) is typically considered the gold standard for methylation status interrogation on a genome-wide scale. As the name suggests, WGBS involves sodium bisulphite treatment of DNA followed by NGS, allowing for single-nucleotide resolution of 5mC patterns [120]. Targeted approaches have also been developed to reduce the cost and resources associated with WGBS by isolating a relevant portion of the genome for subsequent sequencing (Table 2). Briefly, targeted approaches include methyl CpG binding domain (MBD)-isolated genome sequencing (MiGS), where MBD precipitation of genomic DNA isolates regions of high methylated CpG density [121]. Reduced representation bisulphite sequencing (RRBS), which makes use of methylation-sensitive restriction enzymes (MSREs) to recover genome-wide CGIs [122], is also frequently used. For a comprehensive review of targeted approaches, see [123]. WGBS has also been successfully applied to single cells, detecting approximately 48% of CpG sites in the human genome [124]. Though the coverage of this and other single-cell WGBS techniques [125] remains to be improved, they provide exciting opportunities for further characterization of epigenetic heterogeneity in cancer cells. WGBS has also recently been applied to archival bone marrow smears from patients with ALL, opening the door to longitudinal epigenetic studies and investigation of temporal tumour heterogeneity [126].

Though 5mC is the most well characterized DNA modification, oxidized variants of 5mC generated by ten eleven translocation (TET) enzymes, namely 5-hydroxymethylcytosine (5hmC), 5-formylcytosine (5fC), and 5-carboxylcytosine (5caC), can also be found at low frequency throughout the genome [120,127]. Improvements in methods used to quantify and characterize these variants have led to a better understanding and appreciation of their role in cell regulation [120,127]. Conventional bisulphite sequencing cannot distinguish between 5mC and 5hmC, as both variants are resistant to deamination; conversely, 5fC and 5caC are not resistant to bisulphite treatment, but can be converted to resistant residues either by chemical modification or through reduction of 5fC [120]. Methods that allow for relatively quick and inexpensive genome-wide analyses of these variants, such as DNA immunoprecipitation followed by NGS (DIP-seq), chemical labeling, and PCR-based methods are currently being developed and improved (for a review of these methods, see [120]). Single-base pair detection of these variants, however, requires additional resource-intensive methods based on bisulphite sequencing. Oxidative bisulphite sequencing (oxBS-seq) [128] or TET-assisted bisulphite sequencing (TAB-seq) [129] can be used for 5hmC, while chemical modification-assisted bisulphite sequencing (CAB-seq) [130] can be used for 5caC, and fCAB-seq [131] and reduced bisulphite sequencing (redBS-seq) [132] can be used for 5fC (Table 2). For all of these methods, a comparison to WGBS results is needed to differentiate between 5mC and the variant of interest, thereby doubling the amount of sequencing needed (Plongthongkum *et al.* [120] provide an excellent review of these approaches). Another major caveat of these approaches lies in the read depth needed to produce quantitative results, which is extremely high (usually >1000×) due to the variants’ low abundance throughout the genome [133]. Until such drawbacks are addressed, genome-wide, single-nucleotide resolution maps of these variants will continue to be too cost- and resource-intensive to be obtained on a regular basis through conventional sequencing methods. As mentioned above, third-generation sequencing methods are generally able to distinguish between unmethylated cytosine and one or more variants, and hold great promise for the future of genome-wide, single-nucleotide resolution sequencing of DNA modifications [60].

**Table 2:** NGS-based assays used to study the epigenome.

Acronym	Full Name	Target	Reference(s)
WGBS	Whole-genome bisulphite sequencing	5mC	[134]
MiGS	MBD-isolated genome sequencing	5mC (regions of high methylated CpG density)	[121]
RRBS	Reduced representation bisulphite sequencing	5mC (CpG-rich genomic fragments)	[122]
DIP-seq	DNA immunoprecipitation sequencing	DNA modification of interest	[135,136,137]
oxBS-seq	Oxidative bisulphite sequencing	5hmC	[128]
TAB-seq	TET-assisted bisulphite sequencing	5hmC	[129]
CAB-seq	Chemical modification-assisted bisulphite sequencing	5caC, 5fC (fCAB-seq)	[130,131]
redBS-seq	Reduced bisulphite sequencing	5fC	[132]
ChIP-seq	Chromatin immunoprecipitation sequencing	Histone modification/histone variant/DNA-binding protein of interest	[138]
MNase-seq	Micrococcal nuclease sequencing	Nucleosome-associated DNA	[139]
DNase-seq	Deoxyribonuclease sequencing	DNase I hypersensitive sites	[140]
5C	Chromosome conformation capture carbon copy	Spatial distances between genomic regions of interest	[141]

#### 4.3.2. Histone Modifications

In addition to DNA methylation, histone modifications patterns are also often deregulated in cancer, leading to aberrant gene expression and various cancer-related phenotypes. Histone modifications consist of covalent and reversible modifications of core histone proteins, most often by acetylation, phosphorylation, methylation, and/or ubiquitination [142]. The location and composition of the modifications help regulate chromatin accessibility, and therefore influence chromatin-based processes such as DNA transcription and repair [142]. More than a decade ago, Nguyen *et al.* [143] first demonstrated that aberrant histone modifications were likely to play a role in oncogenesis, showing an association between H3K9 hypermethylation and H3K4 hypomethylation and reduced expression of tumour suppressor genes in four cancer cell lines. Indeed, histone modifications are often associated with DNA regulatory elements, and their aberrant localization and abundance can lead to oncogenic deregulation of gene expression [144]. For instance, ~60% of high-grade paediatric gliomas harbour a recurrent K27M mutation in *H3F3A*, one of two genes that encode the histone H3 variant H3.3 [145]. H3K27 is conserved among all H3 proteins, and can be methylated or acetylated in different contexts: in humans, H3K27ac is found at gene enhancers, while H3K27me3 is enriched at silent gene promoters. The *H3F3A* K27M mutation has been shown to lead to a global reduction in H3K27me2 and H3K27me3 levels and to local increases in H3K27me3 and EZH2 (the catalytic subunit of H3K27 methyltransferase), the sum of which results in increased expression of cancer-associated genes [145]. Histone modifications also function in cooperation with DNA methylation, and each can influence the location and composition of the other. For instance, a recent report used data from the Roadmap Epigenomics Consortium project [146] and ENCODE [147] to identify recurrent gene promoter hypomethylation events in GBM that co-occur with H3K4me3 events [148]. They showed that the transcription of affected genes was altered and that, in at least one case, the modifications led to the expression of an oncogenic protein.

The introduction of the chromatin immunoprecipitation (ChIP) method in 1999 [149] represented a turning point for histone modification studies by allowing for the isolation of specific histone modifications along with the fragment of DNA to which the histone is bound. Today, ChIP is combined with NGS (ChIP-seq) in order to sequence these DNA fragments and map them back to a reference genome, providing a genome-wide view of sequences bound by the histone modification of interest [138]. Though ChIP-seq reduces biases associated with earlier array- or PCR-based techniques, its resolution is relatively low due to the large DNA fragments produced by random shearing of the DNA crosslinked with the immunoprecipitated protein of interest [138]. The method is also sensitive to several factors such as antibody specificity and DNA shearing, the latter of which is not generally highly reproducible. ChIP-exo is a modification of the method that uses an exonuclease enzyme to digest the DNA fragments, thereby improving the resolution with which binding sites can be identified [150,151]. An updated ChIP-exo protocol, named ChIP-nexus (chromatin immunoprecipitation experiments with nucleotide resolution through exonuclease, unique barcode, and single ligation), was also recently published that reduces the amount of starting material needed compared to ChIP-exo [152]. Importantly, ChIP-seq can also be used to map the binding sites of DNA-binding proteins (e.g., transcription factors), as well as the localization of histone variants (e.g., H2A.Z) in addition to histone modifications, all of which participate in the regulation of gene expression [153].

#### 4.3.3. Chromatin Organization

Additional aspects of chromatin organization can also have significant effects on the regulation of gene expression. For instance, euchromatin comprises regions of the genome that are actively transcribed and lightly stained by cytogenetic techniques, while heterochromatin is composed of highly condensed and stainable material associated with silent genes [154]. In 2011, two groups reported that long-range DNA interactions and higher-order chromatin architecture predict the landscape of somatic copy-number alterations (SCNAs) in cancer genomes [155,156]. Indeed, a recent study recently found that chromatin features from the cell of origin are better predictors of a tumour’s mutation profile than chromatin features from matched cancer cell lines, indicating that chromatin organization in the cell of origin plays an important role in shaping the future mutational profile of tumour cells [157]. An additional report also showed that, in three colorectal adenocarcinomas, approximately one third of the genome exhibited substantial DNA demethylation compared to normal mucosal samples, and that these regions corresponded to higher-order domains that are indicative of large-scale chromatin decondensation [158]. Importantly, some genes within these regions showed hypervariability of expression within the tumours, which the authors suggested could contribute to tumour cell heterogeneity and associated resistance mechanisms. Though higher-order chromatin organization has been studied less extensively than genomic, transcriptomic, and even other epigenetic aberrations in the context of cancer, it is becoming evident that the three-dimensional structure of chromatin can play an important role in cancer initiation and progression. For a review of higher-order chromatin organization studies, see [159].

DNA digestion with micrococcal nuclease (MNase) or deoxyribonuclease (DNase) followed by sequencing of the resulting fragments can be used as a measure of nucleosome occupancy and chromatin accessibility, respectively [160]. Nucleosome-associated DNA is protected from MNase digestion, which preferentially cleaves “linker” DNA positioned between nucleosomes; therefore, sequencing of undigested DNA allows for the construction of a genome-wide nucleosome map [139,161]. Conversely, DNase I hypersensitive sites are nucleosome-depleted regions of DNA that are more susceptible to digestion by DNase I. Attachment of biotinylated linkers to DNase I-digested fragments thus enables their isolation and subsequent sequencing, thereby allowing for mapping of DNase I hypersensitive sites (*i.e.*, regions of high chromatin accessibility) [140,162]. The three dimensional structure of chromatin within the nucleus has also been shown to influence gene expression and can be probed using a variety of chromosome conformation capture (3C)-based assays. These assays make use of formaldehyde fixation to cross-link sections of the genome that are in physical contact through DNA-bound proteins [163]. The frequency and location of these interactions can then be assessed to provide information about general nuclear organization and chromosome conformation. Chromosome conformation capture carbon copy (5C), is the first 3C-based assay to make use of NGS, and allows for high-throughput studies that measure spatial distances between many regions of the genome [141].

#### 4.3.4. Non-Coding RNAs

Non-coding RNAs, as mentioned above, are increasingly understood to play an important role in epigenetic regulation. Several lncRNA species in particular have been shown to guide epigenetic complexes to their genomic targets, and the deregulation of one or more lncRNA species is involved in many types of cancer. Elevated HOTAIR levels in particular have been reported in several cancers, and have been linked to metastasis and poor prognosis due to its regulatory role in invasive and proliferative phenotypes [164,165]. HOTAIR is involved in the regulation and targeting of Polycomb repressive complex 2 (PRC2), which is a histone methyltransferase that targets H3K27 [166]. HOTAIR is also involved in the regulation of other signalling pathways, such as the Wnt/β-catenin pathway [167], and its aberrantly high expression has been shown to trigger EMT and to help cancer cells acquire a stemness phenotype [168]. Similarly, some miRNAs target epigenetic regulators, their aberrant expression thus leading to further epigenetic dysregulation. For instance, a recent report shows that a novel miRNA over-expressed in DLBCL (miR-10393-3p) is significantly anti-correlated with the expression of *KMT2D* and *EP300*, two genes that are involved in chromatin regulation and that are recurrently mutated in non-Hodgkin lymphoma (NHL) [104]. Several reviews further cover the role of lncRNAs, miRNAs, and other ncRNAs in cancer, many of which function through regulation of epigenetic mechanisms [102,106,169].

### 4.4. Integrative Analyses

Though individual “omics” studies are informative on their own, integrative analyses that incorporate genomic, transcriptomic, and/or epigenetic information can be extremely powerful. For instance, integration of DNA and RNA sequencing data has recently been shown to help increase the sensitivity of variant detection, especially in low purity tumours [170]. Indeed, the software developed by the authors was able to detect up to >100% increases in mutation rates for certain genes in breast and lung cancers compared to studies published by TCGA. Within the cohorts used for the study, the new method identified a higher number of patients with mutations in genes that could theoretically be therapeutically targeted (e.g., *PIK3CA* and *ERBB2*), suggesting that integration of RNA sequencing information and the resulting increased mutation detection sensitivity may be relevant in a clinical setting when determining optimal treatment options. Classification efforts have also greatly benefitted from genomic and transcriptomic analyses and from studies that take multi-platform data into account. TCGA’s comprehensive characterization of gastric adenocarcinoma, for example, revealed four subtypes based on mutation, copy number, gene expression, and methylation data [171]. Though the subtypes were not found to affect survival, they are characterized by genomic features that may help guide the development of targeted therapies [171,172]. Indeed, molecular classification efforts provide valuable diagnostic, prognostic, and therapeutic insights that can be better used to categorize patients and provide them with optimal treatment options. Medulloblastomas, for instance, were initially described as small round blue cell tumours of the cerebellum; however, the discovery of *hSNF5/INI1* mutations in atypical teratoid/rhabdoid tumours (ATRTs) and C19MC amplifications in ETMRs have led these tumour types to be recognized as different diseases [173]. In addition, true medulloblastoma tumours are now classified into four distinct subtypes that differ in their genomic and transcriptional profiles, demographics, and outcomes, and which can be targeted using different therapies [173].

Tumour heterogeneity studies have also greatly benefitted from integrative NGS analyses. Shah *et al.* [174] were the first to use NGS to interrogate clonal diversity and evolution, and did so at the genomic and transcriptional levels in the context of breast cancer progression. They found that primary tumour samples were more likely than metastatic tumours to reveal true tumour-initiating mutations, given that significant evolution occurred with disease progression and the emergence of metastatic tumours, leading to a higher number of mutations in the latter. They also identified six mutations with intermediate frequencies of 1%–13%, indicating that their presence was restricted to minor subclones and confirming that the tumours were composed of genetically distinct clonal populations. Though it has been established that intra- and inter-tumour heterogeneity have critical implications in diagnosis, prognosis, and treatment of various cancer types, progress remains to be made on how best to address the various problems posed by these realities, such as treatment resistance. NGS of single cells and bulk tumour samples are routinely used to study and characterize tumour heterogeneity [79,96,175,176,177,178], and will likely continue to provide valuable insights into evolutionary and resistance mechanisms of specific tumours. Recently, Macaulay *et al.* [179] introduced G&T-seq, a method wherein the genome and the transcriptome of single cells are sequenced in parallel, allowing for inferences to be made regarding the consequence of genetic abnormalities. Such integrated “omics” analyses of single cells are becoming more accessible, and their implementation will help further elucidate cellular heterogeneity mechanisms in cancer.

Large-scale projects such as TCGA and the ICGC have greatly facilitated integrative analyses by providing multi-platform data for hundreds of tumours across major cancer types. Indeed, sample collection for TCGA ended in 2013, with samples from 11,000 patients across 33 tumour types [180]. The success of the pilot phase, which aimed to characterize the genomic and molecular features of GBM and ovarian carcinoma [181,182], led to an expansion of the project and an increase in the use of NGS technologies. Whole-exome sequences have now been obtained for each participant, and whole-genome sequences are available for a subset of participants [105]. RNA-seq data is also available for each of the 33 tumour types, and miRNA-seq data for all tumour types except GBM. Importantly, most of these data sets are made publicly available. The majority of tumour types (25 of 33) have data from >100 cases, with up to 1098 samples for breast cancer. Integrative, comprehensive analyses have been published by TCGA for fifteen individual cancer type studies to date [171,181,182,183,184,185,186,187,188,189,190,191,192,193,194].

#### Pan-Cancer Studies

Both TCGA and other groups have also conducted studies that focus on more than one cancer type, such as TCGA’s pan-cancer analysis project that aims to integrate genomic, transcriptomic, and epigenomic data from several cancer types [195]. In 2014, Hoadley *et al.* [196] published an integrative analysis of multi-platform data from the first 12 cancer types studied by TCGA. Their analysis led to a classification of 11 integrated subtypes, some of which present a near perfect relationship with the tissue of origin and some of which are comprised of different cancer types (e.g., the squamous-like subtype is composed of most head and neck squamous cell carcinomas (HNSCCs) and lung squamous cell carcinomas (LUSCs), some bladder adenocarcinomas, and a few lung adenocarcinomas). This reclassification was significantly better associated with disease outcomes than the classic tissue-of-origin taxonomy, and suggested that a substantial amount of patients would benefit from non-standard treatment regimens. Interestingly, bladder cancers were separated into three subtypes in the new taxonomy system, and survival was correlated with subtype membership. These subgroups were subsequently shown to differ in copy number alterations, protein expression (e.g., HER2, Rab25, and markers of EMT), and immune cell signatures. These and other results reported in the study pose important questions as to how cancer as a whole converges onto specific oncogenic mechanisms, and whether a new cancer-wide classification system may provide more relevant diagnostic and prognostic criteria for clinical trial patient selection and overall treatment options.

In addition to improvements in classification, TCGA and similar multi-platform analyses have also helped reveal new aspects of tumour biology. For instance, a recent study reported a novel tumour suppressor role for *ZBTB7A*, whose transcripts are frequently less abundant in solid tumours, and whose protein product acts as a transcriptional repressor of the critical glycolytic genes *GLUT3*, *PFKP*, and *PKM* [197]. Importantly, the authors found that reduced *ZBTB7A* expression was associated with later stages of cancer and poor patient survival across cancer types, but also conferred a higher sensitivity to glycolysis inhibition, which could be exploited to develop new treatment options. Given that some events may occur at a relatively low frequency within cancer types, but be recurrent within, for example, advanced solid tumours, such a study once again demonstrates the utility of looking beyond traditionally defined cancer types.

Though our understanding of cancer mechanisms has increased dramatically in the past few decades, most cancers, and especially metastatic events, remain difficult to treat and present a high mortality rate. As evidenced by the research done to date, small-scale cancer studies simply do not provide enough information to address the disease in a comprehensive fashion. Large-scale, multi-platform studies, however, provide us with an unprecedented wealth of information and the background knowledge needed to make small-scale studies valuable and informative, as discussed in more detail below.

## 5. Emerging Opportunities in Translational Research and Personalized Approaches

One of the most valuable aspects of NGS is the fact that it can produce an unparalleled amount of data relatively quickly and inexpensively, allowing for comprehensive projects at a scale that would otherwise not be feasible. A wealth of information can be recovered from a relatively small portion of tissue, providing researchers with unprecedented access to the human cancer genome. Importantly, multi-platform studies can also be conducted, allowing us to examine the interplay between DNA mutations, RNA expression, and epigenomic patterns, obtaining a comprehensive overview of cancer cells. Large-scale projects such as TCGA and the ICGC have already made available data from thousands of tumours across major cancer types, and have helped us refine classification systems as well as our general understanding of cancer biology. This has in turn allowed us to begin taking a more personalized approach to cancer research and treatment, as a single tumour can now be studied in the context of the knowledge we have acquired regarding its cancer type and/or subtype, along with general mechanisms of cancer initiation and progression. Indeed, the concept of precision medicine has been gaining traction in the past years, especially in the field of oncology where a tumour genome can be compared to its matched normal genome in order to characterize the cancer and identify targetable modifications. In-depth characterization of individual tumours is therefore of significant potential benefit to patients, whose treatment can chosen based on molecular alterations found within their tumour(s). Such studies can also reveal critical aspects of tumour biology, contributing to our more general knowledge of tumour mechanisms and behaviour. For instance, the Personal OncoGenomics (POG) initiative at the British Columbia Cancer Agency is a project that aims to gauge whether in-depth genomic data can be successfully used to guide clinical decision-making while also cataloguing genomic and transcriptomic information for hundreds of cancer patients [198,199,200,201]. Though this project is, to our knowledge, the only one that addresses precision oncology measures at the whole-genome scale, other groups have also published similar reports. Published case studies from the POG project and from other institutions, summarized below, are enlightening examples of the benefits afforded by precision approaches, illustrating the broad potential of this approach to multiple tumour types.

Jones *et al.* [202] were the first to use genome-wide analysis of a patient’s tumour genome and transcriptome to guide oncology treatment decision-making. Analysis of the primary tumour suggested that it was driven by the *RET* oncogene, offering a rationale for treatment with RET inhibitors. Though the treatment was initially successful, new lesions appeared after seven months. Genomic and transcriptomic analyses of a recurring metastasis detected evidence of activation of the MAPK and AKT pathways, suggesting mutational evolution in response to drug selection and a possible treatment resistance mechanism. A subsequent POG report described how sequencing of a metastasized sphenoid mass originally considered an undifferentiated squamous cell carcinoma allowed for its re-diagnosis as a *SMARCB1*-negative rhabdoid tumour [198]. The new diagnosis prompted a change in therapy, which initially produced positive results. Similarly, Welch *et al.* [203] presented a case where conventional tests could not determine whether the patient had acute promyelocytic leukemia (PML) or AML with cytogenetics indicative of a poor prognosis. The authors sequenced the patient’s genome and were able to identify a cytogenetically cryptic event leading to a *PML-RARA* fusion indicative of PML. The patient, who was originally referred to the centre for treatment with an allogeneic stem cell transplant, was instead treated with all-*trans* retinoic acid (ATRA), and remained in first remission at the time of publication 15 months after presentation. Another group used an in-house-developed panel to identify a BRAF V600E mutation in a patient with intrahepatic cholangiocarcinoma (ICC) [204]. A multidisciplinary tumour board identified the patient as an ideal candidate for dual BRAF and MEK inhibition, the first described instance of this treatment in ICC. At the time of publication, 34 weeks following the beginning of therapy, the patient continued to show response and remained nearly asymptomatic. In the most recent POG study, Sheffield *et al.* [199] reported the characterization of clinically and molecularly dissimilar peritoneal mesothelioma tumours from two patients. This study generated the first whole-genome sequencing data for this rare, relatively poorly understood cancer type. The authors found evidence for the involvement of tumour suppressor genes such as *CDKN2A* and *NF2*, and also revealed novel potential prognostic factors. For instance, one of the patients presented with sarcomatoid histology and multifocal disease, leading to a prediction of poor prognosis; however, the patient responded particularly well to platinum-based chemotherapy, and remained disease-free at the time of publication. The tumour in question was characterized as somatically hypermutated, which has previously been associated with platinum sensitivity in patients with high-grade serous ovarian cancer [205]. This suggests that a hypermutated phenotype of peritoneal mesothelioma might exist and be associated with favourable prognosis. Overall, these studies highlight both the feasibility and the value of precision approaches in oncology, demonstrating significant clinical and research benefits.

The number of studies focusing on patients that respond particularly well to treatment, termed exceptional responders, is also mounting. Tumours that respond exceptionally well to a treatment course, it is reasoned, must harbour unique features that underlie this exceptional response. Such insights may be biologically significant, and may also provide important clues as to how treatment can be improved for normal responders. For instance, tumours with activating *mTOR* mutations [206] or inactivating *TSC1* and *NF2* mutations [207] have both been shown to be sensitive to the mTOR inhibitor everolimus. This indicates that patients who harbour such mutations could be strong candidates for everolimus treatment, and that patients without these mutations may further benefit from alternative therapies. Similarly, Al-Ahmadie *et al.* [208] reported on a patient with metastatic small-cell cancer who achieved a complete and durable response to combined checkpoint kinase 1 (CHK1) inhibition and DNA-damaging chemotherapy. Genomic sequencing revealed a clonal hemizygous mutation in *RAD50*, a member of the Mre11 complex involved in double-stranded break repair. Combined RAD50 hypomorphism and checkpoint inhibition led to attenuation of both the ATM and ATR axes of the DNA damage response (DDR), thereby conferring extreme sensitivity to DNA-damaging agents. The authors point out that ATM and Mre11 complex mutations occur in various cancer types, providing the opportunity to manipulate this sensitizing interaction in various contexts for clinical benefit. In fact, Weber and Ryan [209] suggested that ATM and ATR can serve as therapeutic targets either as a monotherapy in cancers with DDR defects, or as a combinatorial therapy in other relevant cancers. Outlier responses can thus also reveal synthetic lethal interactions, which are defined as two genes that cause cell death if they are both mutated or therapeutically targeted [210]. Such interactions can be exploited to effectively target and kill cancer cells, as a treatment that targets one gene product will only be effective in cells that also contain a mutation in the second gene (*i.e.*, tumour cells). Large-scale or targeted RNA interference (RNAi) screens can also be used to probe for genes that interact with a gene of interest in a synthetic lethal manner. For instance, a recent study made use of a large-scale RNAi screen to identify the anti-apoptotic *BCL-2* gene as being a synthetic lethal partner of the IDH1^R132H^ mutation [211], which is frequently seen in many cancer types, most notably gliomas and AML [212]. Importantly, the authors note that *IDH1*/*2* mutation status may be an important predictor of BCL-2 inhibition response, which has previously been shown to vary between patients.

Many exceptional responder studies are “N of 1” studies that focus on a single patient who shows a particularly unusual response to a therapy that otherwise produces poor results. For instance, the study on everolimus sensitivity mentioned above focused on a single patient who achieved a complete and durable response to the drug while enrolled in a phase II clinical trial that ultimately failed to achieve significant progression-free survival [207]. Though “N of 1” studies are often dismissed as anecdotes, they are increasingly recognized as able to provide meaningful clinical and research results, revealing important mechanisms of drug sensitivity and therapeutic resistance [213]. Indeed, the U.S. National Cancer Institute (NCI) recently launched an exceptional responder programme that aims to collect 100 tumour samples in an effort to identify unifying molecular markers [214,215]. This is in line with published POG case studies, which also highlight the relevance of “N of 1” studies in terms of both patient benefit and research-advancing biological inferences. Furthermore, a new journal named Molecular Case Studies is being launched in October 2015 that will focus on genomic and molecular analyses for precision medicine, both in cancer and in other disease fields [216]. Deep sequencing is of particular consequence in these studies, as it is able to provide significant data from a single sample and allows for comparisons across an ever-expanding set of sequenced cancers.

The continually decreasing cost of NGS is compatible with the notion that patient genomes, transcriptomes, and/or epigenomes may soon be routinely obtained and become accessible for targeted studies, possibly through additional projects similar to the POG initiative and the exceptional responder programme. Decreasing sequencing costs are of particular consequence in regards to sequencing of DNA modifications such as 5mC, which require a higher sequencing depth than regular genome sequencing. We can therefore anticipate continued growth in NGS-driven multi-platform integrative analyses of cancer genomes, which will contribute to an improved understanding of cancer initiation and progression mechanisms, thereby revealing new therapeutic targets and enhancing treatment options.

## 6. Conclusions

The major technological advancements that have been achieved over the past decade, and the use of these to address fundamental problems in cancer research, present an encouraging outlook for the continued application of NGS in an expanding number of different clinical and research settings. Our knowledge of cancer is increasing, leading to options for more effective screening, prevention, and treatment options. NGS technologies have revolutionized the way we study cancer, and have played a significant role in our increased understanding of the disease. Large-scale single- and multi-platform studies in particular have shed light on multiple aspects of cancer biology, from identifying new relevant mutations to characterizing the interplay between the genome, the transcriptome, and the epigenome within the context of different cancer types. This has in turn led to enhanced classification systems and therefore diagnostic and prognostic knowledge, as well as the consideration of targeted therapies that may be deployed in patients with specific molecular alterations. The knowledge generated provides us with the background needed for small-scale studies, including those with an N of 1, to be thoroughly relevant and informative. This is exemplified in published case studies that have provided evidence for the feasibility and relevance of using whole-genome characterization to guide treatment decisions. Importantly, these studies have also shed light on the biology of rare cancer types such as peritoneal mesothelioma, for which genome-wide characterization had not yet been performed. Expectations for the next decade and beyond are high, and it is hoped that the continued evolution of sequencing technologies will help us continue to more successfully manage cancers through more effective screening and monitoring techniques, and to improve therapeutic and curative treatments through targeted treatment.
